# Oxidative Stress in Aquaculture: Pathogenic Mechanisms and Preventive Strategies in Farmed Aquatic Animals

**DOI:** 10.3390/cimb47110873

**Published:** 2025-10-22

**Authors:** Wenjing Ma, Wenting Zeng, Disen Zhang, Yiling Zhou, Yi Huang, Yuhang Hong

**Affiliations:** 1 Key Laboratory of Application of Ecology and Environmental Protection in Plateau Wetland of Sichuan, Xichang University, Xichang 415000, China; 2 Key Laboratory of Animal Disease Detection and Prevention in Panxi District, Xichang University, Xichang 415000, China

**Keywords:** oxidative stress, aquatic animal diseases, reactive oxygen species, antioxidant regulation, aquaculture health

## Abstract

Oxidative stress (OS), defined as a disturbance in the balance between the production and elimination of reactive oxygen species (ROS), has been widely recognized as a key factor in the pathogenesis of various aquatic animal diseases. With the intensification of aquaculture and increasing environmental pressure, aquatic animals are frequently subjected to stressors that trigger oxidative stress, thereby compromising their health and productivity. This review comprehensively summarizes recent advances in understanding the involvement of oxidative stress in multiple organ-related diseases in farmed aquatic animals, including hepatic/pancreatic injuries, gill lesions, muscle degeneration, skin and shell disorders, metabolic disruptions, immunosuppression, and reproductive impairments. The underlying mechanisms involve excessive ROS-induced lipid peroxidation, inflammation, mitochondrial dysfunction, and the disruption of critical signaling pathways. Additionally, recent advances in nutritional antioxidants (e.g., vitamins, plant extracts), environmental regulation, and feed additives for mitigating oxidative damage are also discussed. A comprehensive understanding of the pathogenesis and regulation of oxidative stress is essential for improving aquatic animal health and enhancing the sustainability of aquaculture systems.

## 1. Introduction

In recent years, the rapid expansion and intensification of aquaculture have posed increasing challenges to the health of farmed aquatic animals. High stocking densities, deteriorating water quality, and exposure to pollutants frequently induce stress responses, which compromise immune function, growth performance, and survival rates. Among the many physiological disturbances observed under such conditions, oxidative stress has emerged as a central pathological mechanism linking environmental stress to tissue injury [1,2]. While oxidative imbalance can also occur in wild and ornamental aquatic species, this review specifically focuses on farmed aquatic animals, where intensive aquaculture practices and environmental fluctuations make oxidative stress particularly critical.

**Figure 1:**
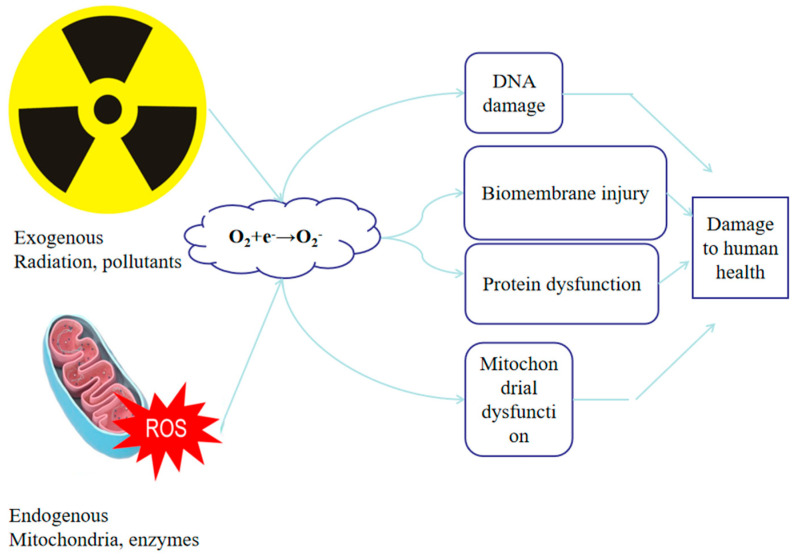
Exogenous and endogenous sources of oxidative stress in farmed aquatic animals. Exogenous stressors, such as radiation, hypoxia, and pollutants, stimulate excessive ROS formation, while endogenous processes like mitochondrial electron leakage, peroxisomal oxidation, and endoplasmic reticulum (ER) stress also contribute to ROS generation. When the antioxidant defense system becomes overwhelmed, ROS can accumulate and trigger cellular damage, including lipid peroxidation, protein oxidation, DNA breakage, and inflammatory signaling. These cellular effects ultimately lead to tissue injury in major organs, metabolic imbalance, and a decline in overall fish health and survival.

In aquatic animals, reactive oxygen species (ROS) encompass several chemically distinct molecules, each differing in reactivity, half-life, and biological function. Superoxide anion (O_2_^−^) is primarily generated during mitochondrial electron transport and serves as the precursor to most other ROS; it exhibits moderate reactivity and can act as a redox signal when rapidly dismutated to hydrogen peroxide (H_2_O_2_) by superoxide dismutase (SOD). H_2_O_2_ has a longer half-life and higher membrane permeability, allowing it to function as a second messenger that modulates cellular signaling pathways, including those governing immunity, metabolism, and stress adaptation. In contrast, the hydroxyl radical (·OH), formed via the Fenton reaction between H_2_O_2_ and transition metals such as Fe^2+^, is extremely reactive and short-lived, causing direct and irreversible oxidation of lipids, proteins, and DNA. Under physiological conditions, ROS act as crucial signaling molecules for cell proliferation, immune activation, and redox homeostasis [3]. However, when ROS generation exceeds antioxidant capacity, pathological oxidative stress occurs, leading to lipid peroxidation, protein carbonylation, mitochondrial dysfunction, and genomic instability [4]. Cellular ROS emerges from both endogenous sources, such as mitochondrial respiration or peroxisomal β-oxidation, and exogenous sources, such as environmental stress including hypoxia, ammonia or exposure to different kind or pollutant (Figure 1) [5,6]. Thus, oxidative stress in aquatic animals represents a dynamic continuum between regulated redox signaling and oxidative injury, rather than a simple “ROS overload” phenomenon.

Under normal physiological conditions, aquatic animals rely on a complex antioxidant system, including enzymatic components such as superoxide dismutase (SOD), catalase (CAT), and glutathione peroxidase (GPx), as well as non-enzymatic antioxidants like vitamin C, vitamin E, and flavonoids, to maintain redox homeostasis [7,8]. However, when aquatic organisms are exposed to environmental stressors, ROS production may increase dramatically. This imbalance initiates a cascade of pathological changes, including lipid peroxidation, mitochondrial dysfunction, activation of proinflammatory pathways, immune suppression, and apoptosis [9,10,11]. These processes are associated with a broad spectrum of diseases that affect critical tissues and organs such as the liver, gills, muscle, and reproductive system [12,13].

Despite increasing research attention on the toxicological effects of oxidative stress, there is a lack of systematic reviews that integrate its role in organ-specific diseases with practical prevention and intervention strategies. Therefore, this paper comprehensively reviews the recent advances in our understanding of oxidative stress-related pathologies in aquatic animals. It outlines the molecular mechanisms underlying oxidative damage across different tissues and discusses current nutritional, environmental, and pharmacological approaches aimed at mitigating its harmful effects. The goal is to provide a theoretical foundation and practical guidance for enhancing oxidative resistance and health management in aquaculture species, ultimately contributing to the sustainable development of the aquaculture industry.

## 2. Oxidative Stress and Aquatic Animal Diseases

### 2.1. Hepatopancreas/Liver Damage

The hepatopancreas or liver plays a central role in the physiological metabolism of aquatic animals, participating in nutrient metabolism, detoxification, and immune regulation [14]. However, with the intensification of aquaculture and the continuous deterioration of the aquatic environment, aquatic animals face increasing environmental stressors, and oxidative stress has become one of the key factors affecting their health and growth [15]. Oxidative stress-induced hepatopancreatic/liver damage not only lowers immunity and increases disease susceptibility but also negatively impacts aquaculture yield and product quality, leading to substantial economic losses [16].

#### 2.1.1. Acute Hepatopancreatic Necrosis Syndrome (AHPNS) in Shrimp

AHPNS is a severe disease that poses a significant threat to the shrimp farming industry [4]. Its main symptoms include hepatopancreatic atrophy, whitening, and the appearance of “white feces” [17]. Studies have shown that oxidized feed lipids and algal toxins are key factors that induce AHPNS. When shrimp consume feed containing oxidized fats, the oxidative byproducts trigger oxidative stress, resulting in excessive ROS production. These ROS attack hepatopancreatic cells, causing lipid peroxidation of cell membranes and compromising cellular integrity [18]. ROS also activate apoptosis-related signaling pathways, leading to hepatocyte apoptosis. In a study on *Litopenaeus vannamei* under cyclic hypoxia (0.8–3.5 mg/L) for 28 days, it was observed that short-term hypoxia enhanced antioxidant capacity, while prolonged exposure impaired antioxidant defense and caused hepatopancreatic damage [19].

#### 2.1.2. Fatty Liver Disease in Fish

**Figure 2:**
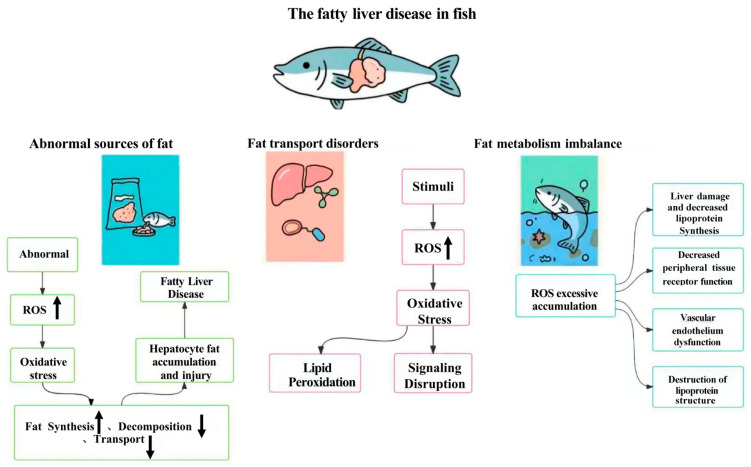
The pathological process of fatty liver disease in fish. This figure illustrates how oxidative stress contributes to the onset and progression of fatty liver disease in fish. Abnormal dietary fat intake leads to excess ROS generation, causing oxidative stress that promotes lipid accumulation and hepatocyte injury. Disruption of fat transport and signaling pathways further enhances lipid peroxidation and metabolic imbalance. Excessive ROS accumulation ultimately impairs hepatic function, decreases lipoprotein synthesis, damages vascular endothelium, and disrupts lipoprotein structure. Together, these processes result in liver dysfunction and systemic metabolic disorders characteristic of fish fatty liver disease. The thin curved arrow indicates biological progress; thick upward arrow indicates upregulation and thick downward arrow indicates downregulation.

Fatty liver disease is commonly observed in cultured species such as largemouth bass [20], grass carp [21], and tilapia [22]. It mainly affects the liver, resulting in hepatic steatosis and significant accumulation of lipids (Figure 2). In severe cases, liver function deteriorates, potentially leading to hepatic lesions and cirrhosis, adversely affecting fish growth and survival. Major causes include high dietary lipid content, imbalanced fatty acid composition, and ingestion of oxidized fats. Prolonged consumption of high-fat diets increases lipid synthesis in the liver, while lipid metabolism and transport capacity remain inadequate, leading to lipid accumulation [21]. This excess fat can induce oxidative stress—ROS generated through lipid peroxidation directly damage hepatocytes—and suppress antioxidant enzymes, further weakening hepatic defenses and exacerbating liver injury. Additionally, oxidative stress alters the expression of genes involved in lipid metabolism, resulting in metabolic dysregulation and progression of fatty liver disease [23].

#### 2.1.3. Other Types of Hepatopancreatic/Liver Damage

Beyond the above-mentioned conditions, oxidative stress is implicated in various other hepatic disorders in aquatic species. For instance, in cadmium-polluted waters, the hepatopancreas of the freshwater crab (*Sinopotamon henanense*) suffers oxidative damage, characterized by altered antioxidant enzyme activity and elevated ROS levels, eventually triggering apoptosis [20]. The mechanism involves cadmium disrupting cellular membranes, reducing cytochrome reductase activity, increasing ROS production, and impairing mitochondrial metabolism. In Indian carp exposed to triclosan (TCS), toxic effects on the liver were observed, with TCS activating the PERK pathway to induce endoplasmic reticulum stress and hepatocyte apoptosis. Experimental data show that TCS exposure significantly elevated ROS levels in a dose- and time-dependent manner, initially over-activating but ultimately decompensating the antioxidant defense system, and altering the expression of ER stress- and apoptosis-related genes [24].

#### 2.1.4. Causality Between Oxidative Stress and Hepatic Injury

Whether oxidative stress serves as a cause or a consequence of hepatopancreatic damage remains context-dependent. Under acute environmental insults such as hypoxia, heavy-metal exposure, or pathogen infection, the excessive generation of ROS frequently acts as an initiating event that directly oxidizes lipids and proteins, triggers mitochondrial dysfunction, and activates pro-apoptotic signaling such as the AHPNS infection in shrimps [4,17]. In contrast, during nutritional imbalance, such as high-fat feeding or exposure to oxidized lipids, oxidative stress may emerge secondarily from lipid overload, endoplasmic-reticulum stress, or inflammatory cytokine release [20]. Multi-omics analyses in shrimp affected by AHPND further support a bidirectional relationship: metabolic disruption and immune activation increase ROS production, which in turn amplifies mitochondrial and endoplasmic-reticulum injury [25]. Therefore, oxidative stress should be viewed as a dynamic component of a feedback loop rather than a single causal event. Initial stressors disturb redox homeostasis, and the ensuing ROS accumulation further accelerates hepatopancreatic pathology, creating a self-reinforcing injury cycle. For example, in hepatic/hepatopancreatic disease, primary etiologies such as Vibrio PirABvp cytotoxicity [15] and cyanobacterial microcystin phosphatase inhibition [26] can initiate lesions upstream of oxidative imbalance; redox pathways (Nrf2-Keap1, MAPK/NF-κB, AMPK, autophagy) are then recruited and amplify damage. Conversely, host factors and legacy exposures modulate oxidative biomarkers at baseline, emphasizing the need to interpret redox endpoints alongside pathogen/toxin load and covariates [27].

#### 2.1.5. Molecular Regulatory Networks of Oxidative Stress

Beyond cellular outcomes such as lipid peroxidation and apoptosis, recent transcriptomic and metabolomic studies have revealed that oxidative stress in aquatic animals involves complex regulatory signaling networks. The Nrf2-Keap1 pathway functions as a master switch controlling the expression of antioxidant enzymes. In *Oreochromis niloticus* exposed to a high-fat diet, hepatic oxidative injury was accompanied by the down-regulation of Nrf2, ho-1, and nqo1 and the up-regulation of keap1, resulting in excessive ROS accumulation and apoptosis [22]. Similarly, dietary emodin in *Megalobrama amblycephala* activated Notch-Nrf2 crosstalk, enhancing sod1 and gpx1 expression and protecting hepatocytes from oxidized-lipid injury [10].

The MAPK/JNK–NF-κB axis is another conserved pathway linking oxidative stress to inflammation and programmed cell death. In tilapia liver, phosphorylation of JNK1/2 and nuclear translocation of NF-κB (p65) triggered up-regulation of caspase-3 and proinflammatory cytokines (tnf-α, il-1β, il-6), which was alleviated by antioxidant supplementation [20]. Comparable activation of MAPK and NF-κB cascades has been observed in crustaceans facing bacterial infection. Multi-omics profiling of *P. vannamei* and *P. indicus* challenged with *Vibrio parahaemolyticus* revealed coordinated enrichment of oxidative phosphorylation, PI3K-Akt, and PPAR signaling pathways, together with altered purine and amino-acid metabolism, demonstrating how redox imbalance reprograms energy metabolism and immune responses [25].

Newly emerging multi-omics data extend these mechanisms to the gut oxidative- metabolic axis. In *Danio rerio* co-exposed to arsenic and nanoplastics, Li et al. [28] showed that combined exposure exacerbated oxidative stress by increasing MDA, SOD, and CAT activities while depleting GSH and GPx. Multi-level integration of metabolomics, microbiota, and oxidative endpoints revealed that gut redox imbalance was tightly coupled with microbial dysbiosis, as well as down-regulation of key metabolic pathways such as glycerophospholipid metabolism, ABC transporters, and ascorbate/aldarate metabolism. These shifts impair membrane integrity and antioxidant capacity, while the up-regulation of autophagy-related pathways suggests a compensatory mechanism to remove damaged organelles.

#### 2.1.6. Preventive and Therapeutic Strategies for Hepatic Oxidative Stress

Proper formulation of aquafeeds to ensure balanced nutrition and reduce intake of oxidized fats is key to preventing oxidative stress. For example, supplementing feeds with appropriate levels of vitamin C and vitamin E can enhance antioxidant capacity and alleviate oxidative stress [29,30]. Vitamin E supplementation significantly increases the activity of SOD and CAT while reducing MDA levels in the hepatopancreas of *L. vannamei*, thereby mitigating oxidative damage. Research also shows that adding puerarin to the diet can promote growth and immunity in largemouth bass (*Micropterus salmoides*) [31]. Moreover, using high-quality feed ingredients and avoiding mycotoxin-contaminated raw materials (e.g., aflatoxins, fumonisins) can reduce oxidative damage caused by free radicals.

Maintaining a healthy aquaculture environment is also critical. Regular monitoring and control of water quality parameters such as temperature, pH, dissolved oxygen, and ammonia are essential. Measures like water exchange and aeration help reduce ammonia levels and its hepatotoxicity. Sediment management and periodic sludge removal also lower the accumulation of harmful substances. In addition, avoiding overstocking and maintaining appropriate stocking density can reduce stress and prevent liver-related diseases [32].

When necessary, hepatoprotective and antioxidant agents may be used. For instance, herbal extracts such as ginsenosides [33] and ginseng polysaccharides [34] exhibit antioxidant, anti-inflammatory, and hepatocyte-regenerative properties. Incorporating these into feeds with high fishmeal substitution improves antioxidative capacity and growth performance in *L. vannamei*. Chemical agents like alpha-lipoic acid also show strong antioxidant activity and can reduce oxidative liver damage. However, careful consideration of dosage, application method, and residue safety is required to avoid adverse effects [35].

### 2.2. Gill and Respiratory System Injuries

Gills are critical organs in aquatic animals responsible for gas exchange, ion regulation, and immune defense. Constantly exposed to the aquatic environment, gills are highly susceptible to various pollutants and adverse environmental conditions. Oxidative stress is a key pathological process in gill injury. When environmental stressors such as high ammonia or heavy metal contamination cause excessive production of reactive oxygen species (ROS) that surpass the organism’s antioxidant capacity, the lipids, proteins, and DNA in gill cells suffer oxidative damage, leading to tissue erosion and functional impairment.

#### 2.2.1. Gill Oxidative Erosion Disease

Gill oxidative erosion is a common condition in fish, characterized by oxidative damage and decay of gill tissue. Major environmental stressors contributing to gill oxidative erosion include high levels of ammonia, nitrite, and heavy metals such as copper and cadmium. High ammonia can penetrate gill epithelial cells, disrupting intracellular pH balance and inducing massive ROS production. Nitrite oxidizes hemoglobin into methemoglobin, reducing oxygen transport capacity and indirectly intensifying oxidative stress. Heavy metals like copper bind to thiol-containing proteins in gill tissues, impairing antioxidant enzyme activity and weakening the ability to scavenge ROS [36].

#### 2.2.2. Black Gill Disease in Crustaceans

**Figure 3:**
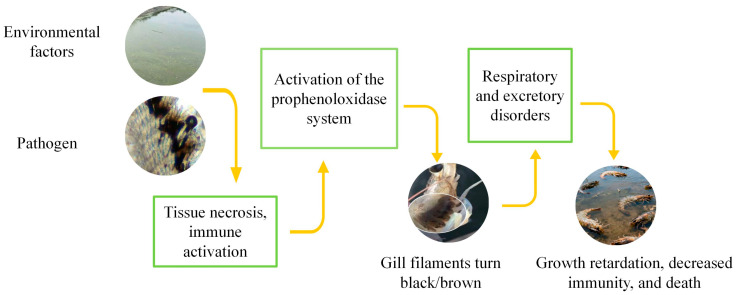
Progress of Black Gill Disease in shrimps.

Environmental stressors, including hypoxia, heavy metals, organic pollutants, or nutritional deficiencies, can induce oxidative stress and elevate ROS levels in gill tissues of shrimp. While ROS may act as signaling molecules contributing to immune activation, the hallmark of black gill disease is melanization in response to pathogenic or environmental insults (Figure 3) [37].

In typical cases of black gill, observed in shrimp like *L. vannamei*, *L. setiferus*, and *F. aztecus*, foreign agents such as ciliates, bacteria or toxins invade the gills, triggering hemocyte aggregation and activation of the prophenoloxidase (proPO) cascade. The resulting phenoloxidase (PO) catalyzes oxidation of phenolic substrates to quinones, which polymerize into melanin and deposit in gill tissues, causing the characteristic blackening [38,39].

#### 2.2.3. Molecular Mechanisms of Oxidative Gill Injury

Excessive ROS attack polyunsaturated fatty acids in gill cell membranes, initiating lipid peroxidation and generating toxic byproducts such as MDA, which compromise membrane integrity. This reduces membrane fluidity and impairs the function of ion transport proteins, leading to dysfunction in gas exchange and ion regulation. For example, under high ammonia stress, the mitochondrial membrane potential in Nile tilapia (*Oreochromis niloticus*) gill cells declines, disrupting respiratory chain function and further amplifying ROS production in a vicious cycle [40].

Gill tissues rely on antioxidant enzymes (SOD, CAT, glutathione peroxidase [GPx]) and non-enzymatic antioxidants (e.g., glutathione [GSH]) to maintain redox balance. In early-stage stress exposure, antioxidant enzyme activity increases compensatorily to eliminate ROS. However, with prolonged exposure, enzyme activity decreases due to exhaustion or structural damage, leading to ROS accumulation and worsening oxidative damage [41].

Oxidative stress activates inflammatory signaling pathways (e.g., NF-κB) in gill tissues, inducing the release of proinflammatory cytokines such as TNF-α and IL-1β, thereby triggering inflammation. Simultaneously, ROS activate mitochondrial apoptotic pathways involving proteins such as Bax and Caspase family members, resulting in epithelial cell apoptosis and compromising gill structural integrity [42].

#### 2.2.4. Prevention and Control Strategies

Preventive strategies include regular monitoring of ammonia, nitrite, and heavy metal concentrations in the water and using biological filters or water conditioners (e.g., zeolite, humic acid) to reduce pollutant levels. Introducing probiotics into the aquatic environment helps regulate microbial communities, reduce ammonia accumulation, and alleviate oxidative stress in gill tissues [43].

In feed, supplementation with antioxidants such as vitamin C, vitamin E, and selenium can significantly improve gill antioxidant capacity. For instance, dietary inclusion of 0.2% vitamin C has been shown to reduce the incidence of black gill disease in *L. vannamei*, increase SOD and GPx activity in gill tissues, and enhance resistance [44].

Immunostimulants such as chitosan and β-glucans can also activate the aquatic animal immune system and indirectly enhance antioxidant defense. Experiments have demonstrated that fish fed β-glucan showed increased LZM activity, phagocytic activity and pathogen resistance [45].

### 2.3. Muscle and Locomotor System Disorders

Muscle tissue in aquatic animals is not only the foundation of locomotion but also serves as a major reservoir for nutrients and plays a crucial role in energy metabolism. Oxidative stress has increasingly been recognized as a key pathological link between environmental stress and tissue damage in muscle systems [46]. When stressors such as oxidized feed, heavy metal exposure, or elevated temperature overwhelm the antioxidant defenses of the organism, excessive reactive oxygen species (ROS) can directly attack muscle cells, inducing lipid peroxidation, protein carbonylation, and apoptosis. These effects lead to muscle whitening, necrosis, and other pathological changes, significantly impairing growth performance and economic value in aquaculture.

#### 2.3.1. Muscle Whitening Disease

**Figure 4:**
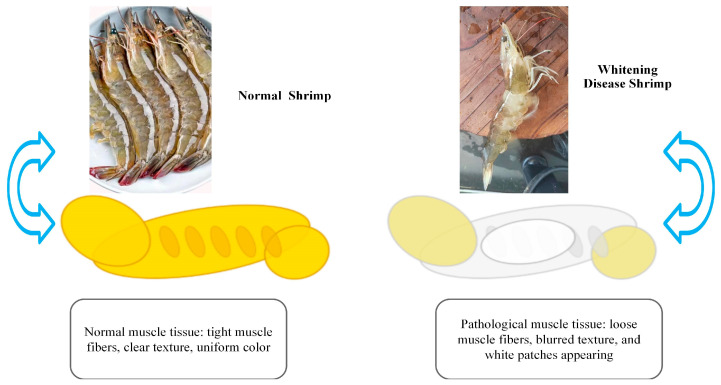
Comparison between normal shrimp and shrimp with muscle whitening disease. Normal shrimp display firm muscle fibers, clear texture, and uniform coloration, indicative of intact mitochondrial structure and balanced metabolism. In contrast, shrimp with whitening disease show loose, blurred muscle fibers with visible white patches, reflecting mitochondrial dysfunction, oxidative stress, and impaired energy metabolism.

Muscle whitening disease is commonly observed in cultured species like shrimp (*L. vannamei*) and tilapia. It manifests as localized or generalized whitening and softening of muscle tissue, and in severe cases, results in loss of mobility (Figure 4) [47]. Studies have indicated that oxidized dietary lipids and environmental pollutants (e.g., polycyclic aromatic hydrocarbons) are major inducers of this condition. Peroxides in oxidized lipids trigger bursts of ROS within muscle cells, leading to significant increases in MDA levels due to membrane lipid peroxidation. Additionally, ROS attack muscle fiber proteins, disrupting their structure and function, which results in fiber breakage and dissolution [48]. In *L. vannamei*, diets containing more than 5% oxidized oils significantly decreased SOD and CAT activity and increased MDA levels in muscle tissues, contributing to noticeable whitening [13].

#### 2.3.2. Molecular Mechanisms of Oxidative Muscle Injury

Muscle cell membranes are rich in polyunsaturated fatty acids, making them vulnerable to ROS attack and subsequent lipid peroxidation. Peroxidation products such as MDA can cross-link membrane proteins, disrupt membrane fluidity and ion channel function, and lead to ionic imbalances that impair muscle contraction. For example, in tilapia exposed to cadmium, lipid peroxidation of muscle cell membranes intensified, while the activity of calcium transport proteins decreased, resulting in muscle relaxation and flaccidity [49].

ROS can also directly oxidize structural muscle proteins such as actin and myosin, leading to carbonylation, cross-linking, or degradation. This disrupts the integrity of the muscle contraction system [50]. Additionally, oxidative modification of antioxidant enzymes (e.g., SOD, GPx) reduces their enzymatic activity, further weakening the organism’s antioxidant defenses and forming a vicious cycle [51].

The energy supply of muscle cells depends heavily on the mitochondrial respiratory chain. Under oxidative stress, mitochondrial membrane lipid peroxidation causes membrane potential collapse and impairs respiratory complex activity, hindering ATP synthesis. Moreover, ROS damage mitochondrial DNA, disrupt mitochondrial biogenesis, and ultimately lead to muscle atrophy or necrosis due to energy deficiency [52].

#### 2.3.3. Preventive and Control Strategies

Using high-quality lipid sources and avoiding oxidized oils in feed is essential. Supplementation with antioxidants such as vitamin C, vitamin E, and astaxanthin can significantly improve muscle oxidative resistance. Studies have shown that increasing astaxanthin content in feed initially enhances weight gain in shrimp, with optimal results at 0.08% inclusion [53].

Water quality management (e.g., aeration, regular water exchange) helps reduce pollutants like ammonia and nitrite, thereby lowering environmental stress. Additionally, the use of probiotics to regulate the gut microbiota has been shown to indirectly enhance muscle antioxidant capacity. For instance, tilapia fed Bacillus subtilis under heat stress exhibited significantly higher muscle antioxidant enzyme activity than controls [54].

Although considerable progress has been made in understanding the mechanisms of oxidative stress-mediated muscle damage, further research is needed to: (1) explore species-specific responses and regulatory networks; (2) elucidate synergistic effects of oxidative stress and other pathological factors (e.g., infections, metabolic disorders); and (3) develop novel antioxidants and precise modulation technologies for early warning and intervention. Future studies integrating single-cell sequencing and metabolomics may help uncover dynamic molecular processes underlying muscle damage, providing innovative solutions for aquaculture.

### 2.4. External Surface and Shell Lesions

Under certain conditions, oxidative stress has been found to correlate with lesions of the external surface and shell in aquatic organisms. For instance, studies have shown that oxidative stress can significantly affect pigment distribution and deposition on the skin of the three-spined stickleback (*Gasterosteus aculeatus*). In one experiment, fish were exposed to potassium dichromate to induce oxidative stress. Skin samples from the dorsal, lateral, and ventral sides were collected during day and night, and melanin cell distribution was used to assess pigment dispersion. Results showed that melanophore counts were significantly higher in nighttime samples. In daytime dorsal skin, oxidative stress caused a notable aggregation of melanin, while no such response was detected in nighttime samples from any skin area [55].

Soft-shell syndrome is a common issue in *L. vannamei* farming and is closely associated with oxidative stress and reduced antioxidant capacity. Studies have found that dietary supplementation with selenium (Se) can effectively alleviate soft-shell symptoms. Selenium is an essential trace element for animals, and selenium nanoparticles (SeNPs), due to their high bioavailability and low toxicity, may serve as promising alternatives to traditional selenium sources (e.g., sodium selenite). In one study, shrimp were divided into five groups and fed diets containing SeNPs or sodium selenite at 0, 0.3, 0.6, and 1.2 mg/kg for eight weeks. Results showed that dietary selenium significantly improved growth performance, shell hardness, and survival rate. Further analysis indicated that selenium enhanced SOD, CAT, and GPx activity in the hepatopancreas and muscle, reduced MDA levels (a marker of oxidative damage), and mitigated oxidative damage in the hepatopancreas and gills in a dose-dependent manner [56].

Additional research has shown that prolonged air exposure in aquatic animals can cause functional hypoxia, disturb intracellular homeostasis, and lead to excessive ROS accumulation. For example, red swamp crayfish (*Procambarus clarkii*) were subjected to air exposure, and their hepatopancreas and gill samples were collected for histopathological observation, antioxidant enzyme activity assay, MDA content measurement, and transcriptome analysis [57]. Results revealed structural changes in gill epithelium and alterations in hepatopancreatic nuclei and mitochondria. Antioxidant enzyme activities initially increased and then declined with exposure duration, while MDA levels significantly rose, indicating enhanced oxidative stress. These changes disrupted enzymes essential for shell synthesis (e.g., phenoloxidase) and interfered with the expression of genes involved in calcium metabolism, thereby impairing shell calcification and reducing shell hardness.

### 2.5. Systemic Metabolic Disorders

**Figure 5:**
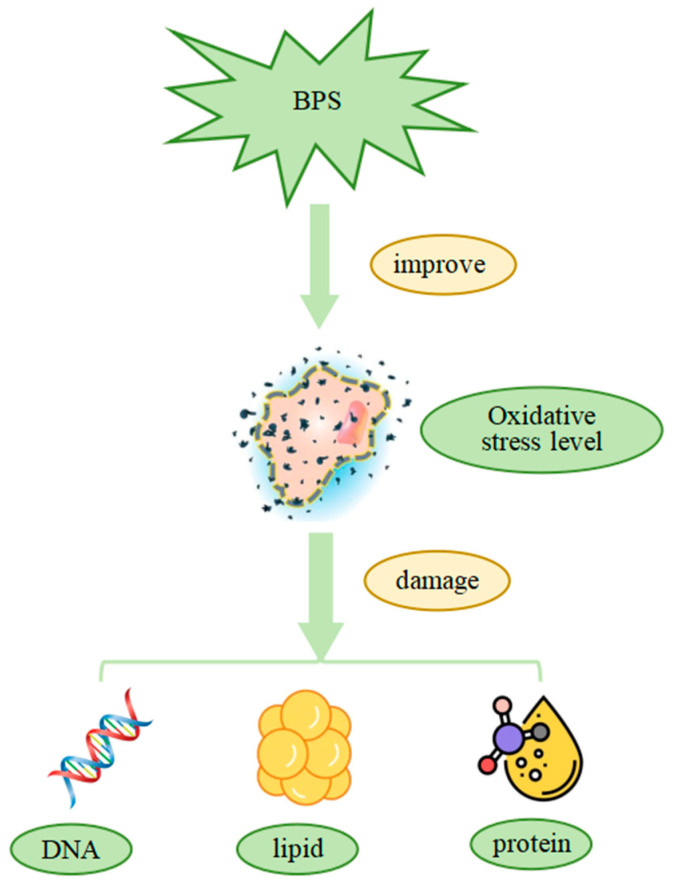
BPS increases the level of oxidative stress and damages biological macromolecules in the body such as proteins, lipids, and DNA.

Oxidative stress has also been implicated in systemic metabolic disruptions in aquatic animals. In a previous study, *P. clarkii* were exposed to various concentrations of BPS solutions. After a fixed exposure period, hepatopancreas tissues—analogous to the mammalian liver and the primary site of metabolism in crustaceans—were collected for biochemical and molecular analyses. Measurements of ROS and MDA levels showed significant increases in BPS-treated groups, indicating oxidative stress and aggravated lipid peroxidation (Figure 5). Enzymatic assays revealed that antioxidant enzyme activities (e.g., SOD, CAT, GPx) initially increased at low BPS concentrations but eventually declined at higher concentrations, suggesting that high-dose exposure overwhelmed antioxidant defenses [58].

Furthermore, indicators of lipid metabolism, such as triglyceride (TG) and total cholesterol (TC) levels, as well as the expression of key lipid metabolism-related genes like fatty acid synthase (FAS) and hormone-sensitive lipase (HSL), revealed signs of lipid metabolic disorders [59]. TG and TC content increased significantly in a dose-dependent manner, indicating enhanced lipid accumulation and disrupted lipid catabolism. Gene expression analysis showed that FAS was upregulated (promoting lipid synthesis), while HSL was downregulated (inhibiting lipid mobilization), resulting in lipid droplet accumulation in hepatopancreatic cells and fatty degeneration, as observed histologically.

Another study found that oxidized fish oil, commonly used in aquafeeds, leads to severe oxidative stress, damaging cell membranes and mitochondria, interfering with enzyme activity and signaling pathways, including activation of CREB1-Bcl2-Beclin1 pathway. This cascade ultimately causes systemic metabolic dysfunction and hinders growth and health [60].

Interestingly, emodin, a naturally occurring compound, was shown to alleviate these symptoms. In an experiment with *Megalobrama amblycephala*, fish were fed diets with different doses of emodin. Liver tissues were then analyzed for MDA, SOD, and CAT activity, as well as gene expression related to the Notch and Nrf2 signaling pathways. The results indicated that emodin downregulated Notch1 while upregulating Nrf2 and downstream antioxidant genes (HO-1, SOD1), thereby enhancing antioxidant capacity and reducing oxidative damage. The antagonistic interaction between Notch and Nrf2 pathways suggests that emodin mitigates oxidative stress by inhibiting Notch signaling and activating Nrf2-dependent antioxidant responses [12].

### 2.6. Immunosuppression and Secondary Infections

The immune system in aquatic animals, like in other vertebrates, plays a crucial role in defending against pathogens such as viruses, bacteria, and parasites by distinguishing self from non-self. It includes both innate and adaptive components, with immune-related organs (e.g., spleen, thymus, kidney) and effector molecules (e.g., immune cells, cytokines, lysozyme) that maintain immunological homeostasis [61,62,63].

Studies have shown that oxidative stress can severely impair immune function. For instance, exposure to heavy metals such as cadmium can significantly increase ROS levels in fish, leading to pathological changes in immune organs like the spleen and head kidney. One study observed a 20–40% reduction in leukocyte counts and a 30–50% decline in macrophage activity in cadmium-exposed fish [64]. Similarly, lysozyme activity, a marker of innate immunity, was found to decrease by 25–50% following short-term cadmium exposure [65]. These findings strongly suggest that oxidative stress disrupts immune homeostasis and function, rendering aquatic animals more vulnerable to infections [66]. Two representative examples of immune suppression associated with oxidative stress in aquatic animals are discussed below: White Spot Syndrome Virus (WSSV) and bacterial septicemia.

#### 2.6.1. White Spot Syndrome Virus (WSSV)

WSSV is a member of the *Nimaviridae* family and causes systemic infections in shrimp and other crustaceans. Infected shrimp exhibit white calcified spots on their exoskeleton and usually die within 3–10 days of infection [67,68]. During the early stages of WSSV infection, the host generates a burst of ROS as part of the innate immune response, particularly via the NADPH oxidase complex in immune cells [69]. This oxidative burst aims to inhibit viral replication by damaging host and viral components [70].

**Figure 6:**
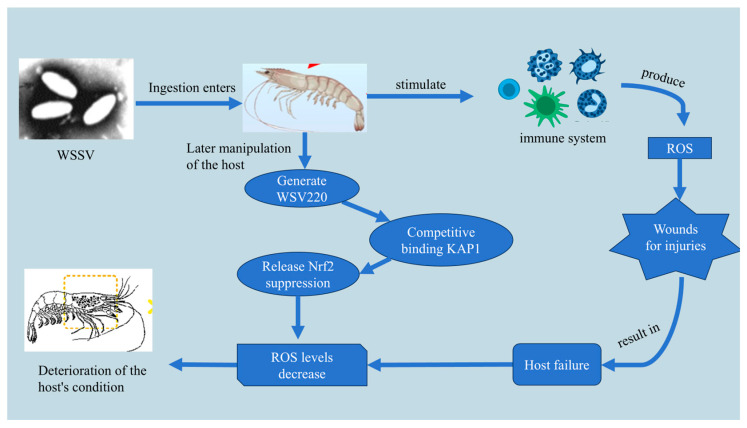
WSSV interferes with immune function through oxidative stress. After ingestion, WSSV invades shrimp tissues and triggers an immune response that initially elevates ROS production. The virus then expresses the protein WSV220, which competitively binds to KAP1 and releases Nrf2 suppression, thereby reducing ROS levels and weakening oxidative defense. This redox imbalance facilitates viral replication, impairs immune function, and leads to tissue injury and progressive host deterioration, ultimately resulting in shrimp mortality.

However, in later stages of infection, WSSV manipulates the host antioxidant system to reduce ROS levels. Specifically, the viral protein WSV220 competitively binds to Keap1 (Kelch-like ECH-associated protein 1), thereby releasing Nrf2 from inhibition. Freed Nrf2 translocates to the nucleus and activates antioxidant gene expression (e.g., G6PDH, SOD, GSH-Px), elevating intracellular GSH and NADPH levels and efficiently eliminating ROS [71]. This viral strategy weakens oxidative defenses and suppresses immune responses, facilitating viral proliferation and host death (Figure 6).

#### 2.6.2. Bacterial Septicemia

*Aeromonas hydrophila* is a facultative anaerobic bacterium widely distributed in freshwater environments. It can cause septicemia in fish, amphibians, and reptiles. Septicemia is characterized by systemic inflammation and immune dysregulation. Increasing evidence suggests that oxidative stress is not only a hallmark of septicemia but also a contributor to its progression [72,73].

**Figure 7:**
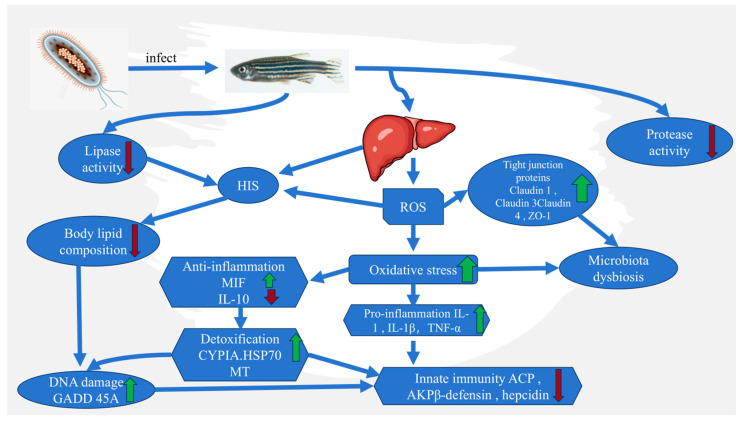
The pathogenesis and effects of bacterial septicemia. Infection elevates ROS levels in hepatic and intestinal tissues, causing tight junction disruption, microbiota imbalance, and lipid metabolic disorders. Oxidative stress induces DNA damage and inflammation (IL-1, IL-1β, TNF-α) while stimulating detoxification (CYP1A, HSP70, MT) and anti-inflammatory responses (MIF, IL-10). These alterations impair liver function and innate immunity, leading to systemic physiological decline characteristic of bacterial septicemia. The green arrow indicates upregulation and red arrow indicates down regulation.

NF-κB is a key transcription factor regulating inflammatory and immune responses. Normally, it is sequestered in the cytoplasm by IκB. Upon stimulation by ROS or pathogen-associated molecular patterns (PAMPs), IKK (IκB kinase) is activated, leading to IκB phosphorylation, ubiquitination, and degradation. This releases NF-κB dimers (e.g., p65/p50), allowing their nuclear translocation and activation of proinflammatory genes [74]. ROS can oxidize IKK and enhance its activity, further promoting IκB degradation and resulting in uncontrolled inflammation. This forms a vicious cycle between oxidative stress and inflammation [75] (Figure 7).

### 2.7. Reproductive and Developmental Impairments

The reproductive development of fish is regulated by a complex interplay of genetic, environmental, and endocrine factors. Environmental stressors such as water pollution, climate change, and heavy metal exposure can induce oxidative stress, thereby interfering with the normal function of the reproductive system. The link between oxidative stress and reproductive decline in aquatic animals has become a significant focus in aquaculture research [76].

Heavy metals are known inducers of oxidative stress. For example, studies have shown that cadmium exposure alters the sex ratio and reproductive development of zebrafish (*Danio rerio*). As cadmium concentration increases, the proportion of male fish declines, and at high doses, ambiguous sex characteristics and developmental malformations are observed [77]. This suggests that oxidative stress may exert damaging effects on gonadal development. On a molecular level, cadmium exposure has been reported to suppress the expression of key reproductive regulatory genes such as nanos, piwi, and dazl, which are essential for germ cell proliferation and migration, resulting in reproductive toxicity [78].

In addition to zebrafish, similar effects have been reported in Nile tilapia. Cadmium exposure triggered inflammatory responses in the gonads, as evidenced by increased expression of proinflammatory cytokines like tumor necrosis factor-α (TNF-α) and interleukin-6 (IL-6). These inflammatory responses further intensified oxidative stress, creating a self-reinforcing loop of damage and dysfunction [79].

From an endocrine perspective, oxidative stress can interfere with the hypothalamic–pituitary–gonadal (HPG) axis, which is crucial for reproductive hormone regulation. Oxidative damage to the hypothalamus can inhibit the release of gonadotropin-releasing hormone (GnRH), while damage to the pituitary gland reduces the secretion of luteinizing hormone (LH) and follicle-stimulating hormone (FSH), both essential for gonadal maturation and gametogenesis [80]. Disruption of the entire endocrine system by oxidative stress may thus impair the normal development and function of reproductive organs [81].

### 2.8. Oxidative Stress Parameters as Biomarkers for Health Monitoring

Oxidative stress parameters serve as sensitive biomarkers for assessing the health status of aquatic animals and predicting disease risks. In practical aquaculture, monitoring these indicators enables early warning and precise management. For instance, to evaluate the stress status of Pacific white shrimp (*Litopenaeus vannamei*) under high-density farming conditions, hemolymph and hepatopancreas samples can be regularly collected for analysis [82]. If the malondialdehyde (MDA) content in the hepatopancreas increases by 40% compared to the previous month, while the activities of superoxide dismutase (SOD) and catalase (CAT) in the hemolymph decrease by 25%, it can be concluded that the shrimp population is under oxidative stress. This often indicates deteriorating water quality (e.g., ammonia nitrogen concentration approaching the safety threshold) or insufficient antioxidant components in the feed, providing a lead time of approximately 2–3 weeks before clinical symptoms such as reduced feeding and growth retardation are observed. Based on this information, farmers can promptly take measures such as increasing aeration, water exchange, or adjusting feed formulations to effectively prevent subsequent diseases like black gill disease or white spot syndrome, significantly reducing economic losses. This monitoring strategy, grounded in oxidative stress indicators, shifts traditional experience-dependent reactive disease treatment to data-driven proactive prevention, representing a critical direction for precision management in modern aquaculture.

### 2.9. Integrated Synthesis Across Stressors

Although the preceding sections discuss oxidative stress under diverse environmental and nutritional challenges, these stressors converge on a limited set of conserved redox pathways that determine tissue vulnerability and repair capacity. Hypoxia, ammonia, and temperature fluctuations primarily impair mitochondrial electron transport, causing excess ROS generation and ATP depletion [13], whereas heavy metals (Cd, Cu, Hg) disrupt thiol-containing antioxidant enzymes and glutathione metabolism, leading to persistent oxidative load [83,84]. In contrast, nutritional imbalances and oxidized dietary lipids provoke endoplasmic-reticulum stress and lipid peroxidation [85], while pathogenic infections and toxins elicit ROS production through immune activation and inflammatory signaling [86]. Despite their differing origins, these insults share activation of Nrf2–Keap1, MAPK/NF-κB, and PI3K-Akt/AMPK pathways, resulting in overlapping outcomes such as apoptosis, autophagy, and metabolic reprogramming. However, each stressor exhibits organ- and species-specific patterns: for example, hypoxia preferentially affects gills and muscle, while dietary and microbial toxins target hepatopancreatic tissues. Multi-omics studies integrating transcriptomics, metabolomics, and microbiota profiling are beginning to reveal how these pathways intersect, allowing the identification of core oxidative modules that underlie adaptive versus pathological responses. Recognizing these cross-stressor commonalities will facilitate the development of generalized antioxidant management strategies and predictive health biomarkers applicable across aquaculture species.

### 2.10. Species-Specific Diversity in Oxidative-Stress Regulation

Although oxidative stress is a universal response to environmental and physiological challenges, the molecular origins of ROS generation and the architecture of antioxidant defenses differ considerably among aquatic taxa, reflecting their evolutionary history and ecological niches. Understanding these differences is essential for translating oxidative-stress research into species-specific health-management strategies in aquaculture.

In mollusks, redox homeostasis is largely governed by canonical enzymatic systems and Nrf2-dependent transcriptional control, but their molecular players can differ from those of vertebrates [87]. For example in the thick-shell mussel *Mytilus coruscus*, exposure to benzo[a]pyrene (BaP) induces the small-Maf homolog McMafF_G_K, which interacts with McNrf2 to up-regulate antioxidant genes such as nqo1 and gpx; silencing McMafF_G_K elevates intracellular ROS and decreases total antioxidant capacity (T-AOC), confirming its regulatory role [88]. Similarly, hypoxia in *Mytilus galloprovincialis* provokes intense lipid peroxidation and the coordinated induction of cat and sod transcripts, suggesting that mussels rely on rapid transcriptional activation of classical antioxidant enzymes rather than extensive signaling cross-talk [89]. These results illustrate that bivalves maintain redox stability primarily through direct enzymatic detoxification and Nrf2-Maf-Keap1 signaling, tuned to their sedentary and filter-feeding lifestyles where intermittent hypoxia and xenobiotic exposure are common.

In crustaceans, oxidative balance is tightly coupled to immune and metabolic processes. A recent study on the shrimp *Exopalaemon carinicauda* exposed to the toxic dinoflagellate *Alexandrium pacificum* revealed marked hepatopancreatic lesions, elevated lipid peroxidation, and suppression of key antioxidant enzymes (SOD, CAT, GSH) [90]. Transcriptomic analyses showed enrichment of differentially expressed genes in endoplasmic-reticulum protein processing, mitophagy, glycolysis/gluconeogenesis, and glycerophospholipid metabolism, linking algal-toxin exposure to concurrent ER stress, mitochondrial dysfunction, and metabolic imbalance. These data illustrate that ROS in crustaceans often originate from both immune-associated NOX activity and ER-stress-induced mitochondrial disruption, with limited antioxidant buffering capacity leading to secondary inflammation and apoptosis.

In teleost fish, ROS generation is mainly metabolic, but regulation is deeply intertwined with nutrient and hormonal signaling. In *Sparus aurata*, transcriptome-proteome-metabolome integration revealed that crowding and hypoxia jointly activate endoplasmic-reticulum stress and suppress insulin-growth-factor signaling, establishing a metabolic–redox coupling that defines the hepatic stress response [91]. Likewise, Ibrahim et al. (2025) [92] investigated Nile tilapia under carbonate-alkalinity stress and found reduced activities of SOD, CAT, and GSH-Px, accompanied by up-regulation of genes involved in PI3K–Akt, MAPK, and Nrf2–Keap1 pathways, indicating redox reprogramming alongside immune and metabolic adjustments. Thus, fish integrate metabolic flux, mitochondrial ROS, and endocrine feedbacks to maintain redox equilibrium, distinguishing their oxidative network from the immune-driven systems of crustaceans or the purely enzymatic detoxification observed in mollusks.

Taken together, these examples highlight that oxidative stress in aquaculture species is taxon-specific in both origin and regulation. Mollusks rely mainly on enzymatic detoxification and Nrf2–Maf signaling, crustaceans emphasize NOX-mediated immune and ER-stress mechanisms, and fish coordinate oxidative balance with mitochondrial metabolism and endocrine cues. Recognizing these mechanistic differences is essential for developing precise antioxidant diets, selective-breeding programs, and stress-mitigation strategies tailored to each aquaculture group rather than applying a single generalized model of “aquatic animal” oxidative stress.

## 3. Prospects

A coherent conceptual framework is needed to link omics discovery, functional validation, microbiome modulation, and sustainable management under the umbrella of oxidative stress. Below, we recast transcriptomics, gene editing, postbiotics, and green aquaculture into such an integrated pathway.

### 3.1. Omics-Guided Discovery of Redox Regulatory Modules

High-throughput transcriptomics, proteomics, and metabolomics are unveiling how aquatic animals respond at the molecular level to diverse stressors. For instance, de Magalhães et al. profiled the liver transcriptome, proteome, and metabolome of *S. aurata* under overcrowding, net handling, and hypoxia, finding that while each stressor elicits distinct gene sets, they commonly converge on downregulation of insulin-growth-factor signaling and induction of endoplasmic reticulum stress [91]. These convergent pathways may represent core redox hubs that mediate cross-stressor resilience.

### 3.2. Gene Editing and Genetic Enhancement of Redox Resilience

Omics-identified candidate genes (e.g., nrf2, gpx, cat, or regulatory kinases) can be functionally tested or even improved using modern genetic tools. In fact, Bayır et al. (2025) review the application of CRISPR/Cas systems in aquaculture and discuss their potential to drive genetic improvement, including traits like disease resistance, stress tolerance, and metabolic efficiency [93]. Meanwhile, Zhu et al. (2024) present a detailed review of CRISPR/Cas9 in fish species, including how precise edits can target growth, immune, or stress-response traits [94]. By applying such techniques to redox-regulatory genes, one could engineer strains with enhanced antioxidant capacity, though off-target effects and ecological safety must be carefully managed.

### 3.3. Microbiome/Postbiotic Interventions as Redox Modulators

Gut microbiota and their metabolites influence host oxidative state via modulation of reactive oxygen species, short-chain fatty acids, and immune interactions. Although direct studies in aquaculture are limited, the framework is promising: postbiotics (non-living microbial products) can serve as stable modulators of redox homeostasis. For example, studies show that postbiotic molecules like exopolysaccharides or cell-wall components can upregulate Nrf2-dependent antioxidant genes and reduce oxidative damage [95]. This suggests that combining microbiome engineering with redox-targeted feed additives may offer a sustainable, non-genetic route to oxidative stress management.

### 3.4. Green and Precision Aquaculture: Applying Redox Knowledge to Sustainable Systems

Translating molecular insights into field practices means embedding real-time oxidative biomarker monitoring, adaptive feed supplementation, and environmental control (oxygen, pollutants, nitrogen load) into a reflexive aquaculture management system. As omics-based biomarkers (Section 2.8) are refined, they can feed into sensor-based decision support systems. This “blue-food” paradigm—linking genetics, microbiome, environment, and management—enables precision oxidative balance, minimizing reliance on chemicals or broad-spectrum treatments.

Altogether, by combining omics-based identification of redox-sensitive targets, gene editing or marker-assisted breeding to reinforce those targets, and postbiotic modulation of gut–host redox ecology, one can envision a unified pipeline from molecular insight to applied oxidative-stress management in aquaculture.

## 4. Conclusions

Oxidative stress, defined as an imbalance between reactive oxygen species (ROS) generation and antioxidant defense, plays a central but context-dependent role in aquatic animal pathology. Yet, major knowledge gaps remain. First, the causal hierarchy between oxidative stress and organ injury is not always clear: ROS may act as primary initiators under acute toxicant or hypoxia exposure, but as secondary amplifiers under chronic nutritional or infectious stress. Determining these causal thresholds requires time-resolved in vivo imaging and controlled challenge experiments.

Second, although core signaling pathways such as Nrf2-Keap1, MAPK/NF-κB, PI3K-Akt/AMPK, and autophagy are well recognized, their tissue-specific coordination and cross-talk remain poorly characterized. Integrative multi-omics approaches, combining transcriptomics, metabolomics, and microbiota profiling, could uncover regulatory nodes that differentiate adaptive from pathological oxidative responses.

Third, most antioxidant interventions (vitamins, polyphenols, probiotics) have been tested under laboratory conditions, often at doses not representative of commercial aquaculture. Their long-term efficacy, ecological safety, and cost-effectiveness require systematic field validation.

Fourth, oxidative-stress biomarkers used for health monitoring (e.g., SOD, CAT, MDA) are context-sensitive, being influenced by body size, developmental stage, and historical exposure, as highlighted by recent evidence from field species. Developing integrative indices that incorporate physiological and environmental covariates will improve diagnostic reliability.

Finally, the interplay between oxidative stress and pathogen/toxin mechanisms remains an area of active debate, where redox imbalance may both result from and exacerbate primary etiological damage. Clarifying these bidirectional dynamics will require combined infection-omics and experimental-toxicology frameworks.

Addressing these gaps will not only refine mechanistic understanding but also advance precision aquaculture, where oxidative stress management is embedded within sustainable nutrition, water-quality control, and disease-prevention strategies. This review differs from previous species- or disease-specific summaries by providing a comparative overview of oxidative stress across multiple organs and aquaculture taxa, highlighting common redox-signaling hubs and distinct adaptive mechanisms that underlie diverse pathologies.

## Data Availability

No new data were created or analyzed in this study. Data sharing is not applicable to this article.
